# Intelligent Biosignal Analysis Methods

**DOI:** 10.3390/s21144743

**Published:** 2021-07-12

**Authors:** Alan Jovic

**Affiliations:** Faculty of Electrical Engineering and Computing, University of Zagreb, Unska 3, 10000 Zagreb, Croatia; alan.jovic@fer.hr

This Editorial presents the accepted manuscripts for the special issue “Intelligent Biosignal Analysis Methods” of the Sensors MDPI journal.

The special issue consists of 12 accepted manuscripts (11 regular papers and 1 review paper) that present advances in research and development of intelligent biosignal analysis methods. These methods are involved in a variety of significant biomedical applications, ranging from clinical decision support systems and portable personal devices used for screening and monitoring patients to modeling organism states and disorders. The focus of this special issue is on different types of methods used for intelligent analysis, and therefore, the accepted manuscripts mostly use different types of machine learning and deep learning algorithms for classification tasks on different types of biosignals. Both the regular contributions and the review paper reach valuable conclusions and discuss avenues for future work in this exciting field. In continuation of this Editorial, the motivation, methods, results, and conclusions of the accepted papers are presented.

The first paper [1], titled “Multi-Branch Convolutional Neural Network for Automatic Sleep Stage Classification with Embedded Stage Refinement and Residual Attention Channel Fusion”, was authored by T. Zhu, W. Luo, and F. Yu, from Zhejiang University, China.

The paper addresses a machine learning method developed for automatic sleep stage classification, which is an important and currently unsolved problem. An efficient and accurate solution could allow clinicians to efficiently assess a person’s sleep quality and help diagnose a possible sleep disorder. The sleep stage refinement process was integrated into a neural network model to enable true end-to-end processing. Since detection can be made more efficient by incorporating multichannel signals, the authors used Sleep-EDF Expanded Database (Sleep-EDFx) and the University College Dublin Sleep Apnea Database (UCDDB), both of which include electroencephalogram (EEG), electrooculogram (EOG), and chin electromyogram (EMG). A multibranch convolutional neural network (CNN) was combined with a proposed residual attention method, and achieved a classification accuracy of 85.7% and 79.4% on the Sleep-EDFx and UCDDB databases, respectively, for the awake state and four sleep stages (N1, N2, N3, and REM). The proposed residual attention method had a more robust channel-information fusion ability than the respective average and concatenation methods.

The second paper [2], titled “Robust T-End Detection via T-End Signal Quality Index and Optimal Shrinkage”, was authored by P.-C. Su, E.Z. Soliman, and H.-T. Wu, from Duke University and Wake Forest School of Medicine, USA, and from the National Center for Theoretical Sciences, Taiwan.

The authors address the problem of accurately detecting the end of the T-wave for providing automatic annotations in the electrocardiogram (ECG). This problem is difficult to solve due to the presence of noise in ECG signals. The authors propose an algorithm based on the signal quality index (SQI) for the T-wave end (tSQI) and the optimal shrinkage (OS). For segments with low tSQI, the OS is applied to enhance the signal-to-noise ratio. They evaluate their method on a set of 11 short-term ECG recordings from the freely available QT database and on four long-term ECG recordings. They find that tSQI captures signal quality well, and that the proposed OS denoising helps stabilize existing T-end detection algorithms under noisy situations. The methods could be well-suited for clinical applications and large ECG databases; however, the current study is limited by the small size of the annotated datasets available for consideration.

The paper [3], titled “EEG-Based Estimation on the Reduction of Negative Emotions for Illustrated Surgical Images”, was authored by H. Yang, J. Han, and K. Min, from Sangmyung University, Korea.

The paper describes an approach to emotion recognition from EEG biosignals that aims to demonstrate that illustrated surgical images reduce negative emotional reactions compared to that of photographic surgical images. Because surgical images are important in communicating procedures to patients and nonspecialists, it is important to evaluate whether illustrated images that are similarly informative produce the same emotional response. To demonstrate the difference in emotional response, the authors recorded the emotional responses of 40 subjects to 10 pairs of images (surgical images and their illustrated counterparts). They combined their responses with a deep learning network to obtain accurate predictions of emotions. The results show that the estimated valence of illustrated surgical images is significantly higher than the valence of photographic surgical images and vice-versa for arousal, suggesting that illustrated images are better suited for communication with patients and nonspecialists.

The paper [4], titled “Wearable Sensors for Assessing the Role of Olfactory Training on the Autonomic Response to Olfactory Stimulation”, was authored by A. Tonacci, L. Billeci, I. Di Mambro, R. Marangoni, C. Sanmartin, and F. Venturi, from the following institutions in Italy: National Research Council of Italy (IFC-CNR), University of Pisa, and NexFood Srl.

The authors focus on the key psychophysiological drivers of olfactory-related pleasantness, as the current literature demonstrated the relationship between odor familiarity and odor valence but has not clarified the consequential relationship between the two domains. They use analysis of ECG and galvanic skin response at the beginning and end of the olfactory training for 25 subjects enrolled in the study. The authors observed different autonomic system responses, with a higher parasympathetically-mediated response at the end of the training period. They conclude that increased familiarity with the suggested stimuli could lead to a higher tendency towards relaxation, which could be important for applications in personalized treatments based on odors and foods.

The paper [5], titled “Detection of Myocardial Infarction Using ECG and Multi-Scale Feature Concatenate”, was authored by J.-Z. Jian, T.-R. Ger, H.-H. Lai, C.-M. Ku, C.-A. Chen, P.A.R. Abu, and S.-L. Chen, from Chung Yuan Christian University and Ming Chi University of Technology, Taiwan, and from Ateneo de Manila University, Philippines.

The authors investigate different convolutional neural networks (CNN) architectures that can be used for automatic detection of myocardial infarction (MI). Based on data from 200 patients from open-access PTB ECG database, the authors determined that the best network structure can be obtained via tuning both the number of filters in the convolutional layers and the number of input signal scales in the CNN. A multi-lead features-concatenate narrow network (N-Net) achieved 95.76% accuracy on the MI detection task, while multiscale features-concatenate network (MSN-Net) achieved 61.82% accuracy on the MI locating task, which is a much more difficult task than MI detection. Both results outperfomed the state-of-the-art. The authors note that the constructed models are small in size, making them suitable for incorporation into wearable devices for offline monitoring.

The paper [6], titled “EEG-Based Sleep Staging Analysis with Functional Connectivity”, was authored by H. Huang, J. Zhang, L. Zhu, J. Tang, G. Lin, W. Kong, X. Lei, and L. Zhu, from the following institutions in China: Hangzhou Dianzi University, Southwest University, and the Ministry of Education.

Similar to [1], this paper also deals with automatic sleep staging. The authors propose to use indices of functional connectivity from multiple EEG signal channels to classify sleep stages. The main idea of the paper is that brain functional connectivity during a sleep stage process is characterized by different frequency bands and that the interaction between brain regions may be specific to a particular sleep stage. They applied phase-locked value (PLV) to characterize phase synchronization between pairs of signals from different brain regions. The authors demonstrate the effectiveness of using PLV for sleep stage discrimination, which is due to PLV increasing in the lower frequency band (delta and alpha bands) during different stages of nonrapid eye movement (NREM) sleep. They achieved a classification accuracy of feature-level fusion from six frequency bands for NREM, N2, and N3 stages of 96.91% and 96.14% for intrasubject and intersubject evaluations, respectively.

The paper [7], titled “Wearable Technologies for Mental Workload, Stress, and Emotional State Assessment during Working-Like Tasks: A Comparison with Laboratory Technologies”, was authored by A. Giorgi, V. Ronca, A. Vozzi, N. Sciaraffa, A. di Florio, L. Tamborra, I. Simonetti, P. Aricò, G. Di Flumeri, D. Rossi, and G. Borghini, from several institutions in Italy: BrainSigns, Sapienza University of Rome, Ernst & Young, IRCCS Fondazione Santa Lucia, and LUISS University.

The authors contend that consumer wearables are characterized by their ease of use and their comfortability, and therefore, may be an ideal substitute for laboratory technologies in the real-time assessment of human performance in ecological settings. The study evaluated the reliability and capability of two consumer wearable devices, Empatica E4 and Muse 2, to assess stress, mental workload, and emotional state evaluation, while 17 participants performed 3 work-like tasks. The results were compared to that of laboratory devices. The study showed that the parameters computed by the consumer wearable devices and the laboratory sensors were positively and significantly correlated. In addition, wearable devices were shown to be able to discriminate stress levels, but not mental workload or emotional state. 

The paper [8], titled “Constructing an Emotion Estimation Model Based on EEG/HRV Indexes Using Feature Extraction and Feature Selection Algorithms”, was authored by K. Suzuki, T. Laohakangvalvit, R. Matsubara, and M. Sugaya, from Shibaura Institute of Technology, Japan.

This study investigated the use of inexpensive and simple EEG and photoplethysmography (PPG) sensors to classify emotions using EEG and heart rate variability (HRV) signal features. The authors extracted the features and then used feature selection methods to improve the accuracy of the model. Multiple feature combinations of EEG and/or HRV indices selected using feature selection criteria were used to construct emotion estimation models based on a deep neural network. The model was evaluated using the stratified *k*-fold cross-validation method on a dataset consisting of 25 young and healthy subjects. The results suggest that it is possible to construct an emotion classification model using only a small number of features from physiological indices. Using the proposed methodology, an accuracy of approximately 98% was achieved for classification into 4 emotion classes.

The paper [9], titled “The Effects of Individual Differences, Non-Stationarity, and the Importance of Data Partitioning Decisions for Training and Testing of EEG Cross-Participant Models”, was authored by A. Kamrud, B. Borghetti, and C. Schubert Kabban, from the Air Force Institute of Technology, USA.

The study points out the problems associated with dataset construction and partitioning for EEG studies. Due to the nonstationarity of the EEG signal as well as individual differences, EEG-based deep learning models may either not generalize well or, if improperly learned, achieve overly optimistic accuracy. Therefore, it is important that the evaluation procedure strictly follows the evaluation guidelines, as explained in this paper. The data must be partitioned into training, validation, and testing sets so that cross-participant models avoid overestimating model accuracy. The authors build the models for five publicly available datasets and demonstrate how model accuracy is significantly reduced when proper guidelines for cross-participant EEG model are followed. They show that if the guidelines are not followed, the error rates of the cross-participant models can be underestimated by between 35% and 3900%. The authors believe that misrepresentations of model performance in related EEG studies impede scientific progress toward truly more accurate models.

The review paper [10], titled “A Review of EEG Signal Features and Their Application in Driver Drowsiness Detection Systems”, was authored by I. Stancin, M. Cifrek, and A. Jovic, from the University of Zagreb, Croatia.

The work has several contributions. First, it provides a comprehensive overview, systematization, and brief description of the existing features of the EEG signal, including: time-domain, frequency-domain, time-frequency domain, nonlinear, entropies, undirected and directed spatiotemporal, and complex network features. Second, it provides a comprehensive review of drowsiness detection systems reported in the relevant scientific literature. Third, it provides a comprehensive overview of the existing similar reviews, as several related yet different reviews were published in the literature on this topic. Finally, it discusses various ways to improve the state-of-the-art of drowsiness detection systems. Some of the important conclusions reached regarding drowsiness detection systems are: (1) systems should consider raw data, features from all seven categories, and deep learning models; (2) systems should base ground truth labels on the unified, standardized definition and description of drowsiness or be confirmed by multiple independent sources to reduce subjectivity bias; (3) systems should be evaluated on a large number of participants, such as 100 or more, because electrophysiological signals have high interindividual differences. 

The paper [11], titled, “An Improved Deep Residual Network Prediction Model for the Early Diagnosis of Alzheimer’s Disease”, was authored by H. Sun, A. Wang, W. Wang, and C. Liu, from the Northeastern University and the Shenyang University, China.

This paper deals with the early diagnosis of Alzheimer’s disease (AD) from magnetic resonance imaging (MRI) of subjects. The authors use a deep residual network model (ResNet) based on spatial transformer networks and the nonlocal attention mechanism for diagnosing AD. The proposed approach can solve the problems of local information loss and extract the long-distance correlation in MRI feature space, both of which are problematic for conventional convolutional neural networks. The method was evaluated with the Alzheimer’s disease neuroimaging experimental dataset and proved superior to other methods, including the original ResNet model. It achieved 97.1% accuracy, 95.5% macro precision, 95.3% macro recall, and 95.4% macro F1 score. The authors conclude that the proposed method is of great importance for early diagnosis of AD.

Finally, the paper [12], titled “Event-Centered Data Segmentation in Accelerometer-Based Fall Detection Algorithms”, was authored by G. Šeketa, L. Pavlaković, D. Džaja, I. Lacković, and R. Magjarević, from the University of Zagreb, Croatia.

This study addresses the problem of improving algorithms for automatic fall detection. It investigates how different configurations of windows for data segmentation affect the detection accuracy of a fall detection system. The study showed that the best configuration consists of three sequential windows (preimpact: 3.5 or 3.75 s, impact: 0.5 or 1.0 s, and postimpact: 3.5 or 3.75 s). The method was evaluated on three publicly available datasets on falls and activities of daily living using a support vector machine classifier. The achieved F-scores for the public datasets were: 99.7% for ErciyesUni, 96.1% for FallAllD, and 98.4% for the SisFall dataset. The authors conclude that data segmentation is an important component of automatic fall detection systems as it affects the overall detection accuracy. The results of the study clearly suggest that using three windows for data segmentation results in better fall detection performance than using one or two windows.
